# Immunotherapy in HER2-positive breast cancer: state of the art and future perspectives

**DOI:** 10.1186/s13045-019-0798-2

**Published:** 2019-10-29

**Authors:** E. Krasniqi, G. Barchiesi, L. Pizzuti, M. Mazzotta, A. Venuti, M. Maugeri-Saccà, G. Sanguineti, G. Massimiani, D. Sergi, S. Carpano, P. Marchetti, S. Tomao, T. Gamucci, R. De Maria, F. Tomao, C. Natoli, N. Tinari, G. Ciliberto, M. Barba, P. Vici

**Affiliations:** 1 Division of Medical Oncology 2, IRCCS Regina Elena National Cancer Institute, Via Elio Chianesi, 53-00144 Rome, Italy; 2 Department of Clinical and Molecular Medicine, “Sapienza” University of Rome, Azienda Ospedaliera Sant’Andrea, Rome, Italy; 3 HPV-UNIT, UOSD Tumor Immunology and Immunotherapy, Department of Research, Advanced Diagnostic and Technological Innovation (RIDAIT), Translational Research Functional Departmental Area, IRCSS Regina Elena National Cancer Institute, Rome, Italy; 4 Department of Radiation Oncology, IRCCS Regina Elena National Cancer Institute, Rome, Italy; 5 Medical Oncology Unit B, Policlinico Umberto I, Rome, Italy; 6 Department of Radiological, Oncological and Anatomo-Pathological Sciences, Policlinico Umberto I, ‘Sapienza’ University of Rome, Rome, Italy; 7 Medical Oncology, Sandro Pertini Hospital, Rome, Italy; 8 Institute of General Pathology, Catholic University of the Sacred Heart, Rome, Italy; 9 Department of Medical Oncology, Policlinico Universitario “A. Gemelli”, Rome, Italy; 10 Department of Gynecology-Obstetrics and Urology, “Sapienza” University of Rome, Rome, Italy; 11 Department of Medical, Oral and Biotechnological Sciences and Center of Aging Science & Translational Medicine (CeSI-MeT), G. d’Annunzio University, Chieti, Italy; 12 Scientific Direction, IRCCS Regina Elena National Cancer Institute, Rome, Italy

**Keywords:** Metastatic, Early, Breast cancer, Immunotherapy, Vaccine, HER2+

## Abstract

Breast cancer (BC) is a complex disease with primary or acquired incurability characteristics in a significant part of patients. Immunotherapeutical agents represent an emerging option for breast cancer treatment, including the human epidermal growth factor 2 positive (HER2+) subtype. The immune system holds the ability to spontaneously implement a defensive response against HER2+ BC cells through complex mechanisms which can be exploited to modulate this response for obtaining a clinical benefit. Initial immune system modulating strategies consisted mostly in vaccine therapies, which are still being investigated and improved. However, the entrance of trastuzumab into the scenery of HER2+ BC treatment was the real game changing event, which embodied a dominant immune-mediated mechanism. More recently, the advent of the immune checkpoint inhibitors has caused a new paradigm shift for immuno-oncology, with promising initial results also for HER2+ BC. Breast cancer has been traditionally considered poorly immunogenic, being characterized by relatively low tumor mutation burden (TMB). Nevertheless, recent evidence has revealed high tumor infiltrating lymphocytes (TILs) and programmed cell death-ligand 1 (PD-L1) expression in a considerable proportion of HER2+ BC patients. This may translate into a higher potential to elicit anti-cancer response and, therefore, wider possibilities for the use and implementation of immunotherapy in this subset of BC patients. We are herein presenting and critically discussing the most representative evidence concerning immunotherapy in HER2+ BC cancer, both singularly and in combination with therapeutic agents acting throughout HER2-block, immune checkpoint inhibition and anti-cancer vaccines. The reader will be also provided with hints concerning potential future projection of the most promising immutherapeutic agents and approaches for the disease of interest.

## Background

The most relevant predictors of prognosis and treatment outcomes in BC include the expression of hormone receptors (HR) and the overexpression of HER2 or amplification of the inherent gene [1]. Breast cancer is classified on the basis of the presence or absence of these factors into four intrinsic molecular subtypes: luminal A, luminal B, HER2 enriched, and triple negative breast cancer [2], which determine different biological behaviors and diverse clinical evolutions [3]. HER2+ BC constitutes 15–20% of newly diagnosed invasive breast carcinomas [4]. HER2-blocking therapies, such as trastuzumab and/or pertuzumab in combination with chemotherapy represent the standard first-line treatment for HER2+ metastatic (m) BC [5]. In addition, several HER2-targeting therapeutics, including the drug-antibody conjugate ado-trastuzumab emtansine (T-DM1), and, less recently, lapatinib, a reversible tyrosine kinase inhibitor (TKI), have been approved for the treatment of this tumor [6, 7]. However, HER2+ mBC will eventually progress in most patients because of primary or secondary resistance to anti-HER2-directed therapies, including trastuzumab [8]. The impellent necessity for the development of novel therapies and new approaches to overcome the limitations of targeted therapy and improve treatment outcomes oriented our choice on the topic of interest. Indeed, immunotherapy may represent an additional option for HER2+ BC patients, although, thus far, the expansion of the immune-oncology field has found a relatively narrow space in the landscape of breast cancer treatment. When globally considered, breast carcinoma is classified as a moderately immunogenic cancer [9, 10]. However, studies on somatic mutations and tumor microenvironment wherein data analyses are performed by molecular subtypes have shown remarkable heterogeneity [11–13], with the highest immunogenic potential being ascribed to triple negative breast cancer (TNBC) and HER2+ BC among BC subtypes [14]. Considering the efficacy of immunotherapeuticals in highly mutated tumors with high infiltration of immune cells, a relevant benefit from immunotherapy is expected in the HER2+ mBC setting. The inhibition of the hyperactive HER2 protein kinase receptor by trastuzumab in HER2+ breast cancer cells [15] can be partly considered an immunotherapy strategy, since the monoclonal antibody (mAb) mechanism of action includes an immune-mediated component [16]. On this basis, clinical trials have addressed the possibility of enhancing this immune mechanism to overcome the anti-HER2 resistance. In the same optics of exploiting the immune-mediated mechanism of some HER2-blockers, preclinical studies have investigated their potential synergistic effect in association with the cytotoxic T lymphocyte-associated antigen 4 (CTLA4) block or with the programmed cell death protein 1 (PD1) inhibition, demonstrating a robust lymphocytic activation against BC cells [17, 18]. Currently, these combinations are being evaluated in different BC settings. Further promising strategies to capitalize on the immunogenic properties of HER2+ BC include the anti-HER2 vaccine therapies, which have been tested in different formulations in both preclinical and clinical studies.

Herein, we discuss preclinical evidence on mechanistic aspects potential strategies and data from clinical trials on immunotherapy for HER2+ BC. Future perspectives are also envisioned concerning the use of immutherapeutic agents and vaccines in HER2+ BC. The inherent pitfalls and caveats of the most attractive strategies will be also briefly discussed.

## Methods

We searched PubMed from inception to April 2019. We aimed at identifying both intervention trials and observational studies focused on immunotherapy for HER2+ breast cancer. Intervention trials were included independently on the phase and randomized allocation. When considering observational studies, those with a prospective design and a control group were judged suitable for inclusion. Mixed design studies, i.e., observational studies carried out in patients enrolled in intervention trials, were allowed. Evidence from preclinical studies was also considered. References were identified by combining the following terms used both as text words and medical subject headings: immunotherapy, vaccines, HER2-positive, and breast cancer. Once the manuscripts of interest were identified, the reference lists were screened for additional relevant papers. In addition, we consulted the ASCO proceedings from 2010 to 2018.

## Anti-cancer immune response in HER2+ breast cancer and its correlates

**Fig. 1:**
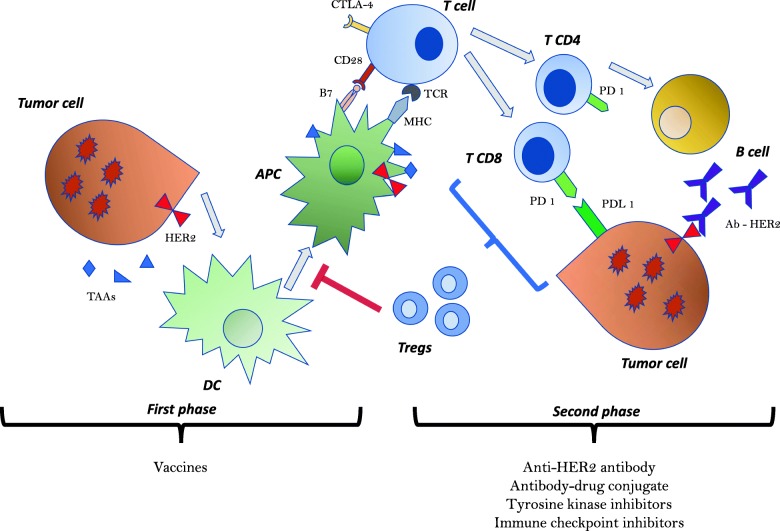
Graphic representation of anti-cancer immune response in HER2+ breast cancer. The anti-cancer immune response is composed by two phases: in the first phase (early phase), DCs sample, process, and present TAAs (such as HER2) generating T cell triggering (both CD4 and CD8) with consequent specific antibody production and cytotoxic cell activation. The process involved in the first phase is responsible for the development of vaccines. The second phase takes place in peripheral areas where specific anti-HER2 antibodies and activated cytotoxic cells explicit their functions. The second phase is the main target of other anti-tumor drugs such as anti-HER2 antibody, antibody-drug conjugate, tyrosine kinase inhibitors, or immune checkpoint inhibitors. Legend: Ab-HER2= anti-HER2 antibody; APC= antigen-presenting cell; B7= B7 protein; CD28= T cell costimulatory molecule CD28; CTLA-4= cytotoxic T-lymphocyte–associated antigen 4; DC= dendritic cell; HER2= human epidermal growth factor 2; MHC= major histocompatibility complex; PD-1= Programmed cell death protein 1; PDL-1= Programmed death-ligand 1; TAAs= tumor-associated antigens; TCR= T-cell receptor; Tregs= regulatory T cells; T CD8= CD8+ cytotoxic T cell; T CD4=CD4+ T cell

The development of an immune response against cancer undergoes different steps broadly encompassed within two major domains, i.e., the early phase, wherein key actors of the immune system are directly involved at the immune system organs’ level, and the late phase, that occurs at the tumor cell site and contemplates the effective anti-tumor response, which contemplates a dynamic interaction with the microenvironment. In the early phase, dendritic cells (DCs) sample tumor-associated antigens (TAAs), such as the HER2 protein [19], and then process and present them after an appropriate maturation signal; otherwise, tolerance will be established [20]. An activated mature DC will then dispatch its role as an antigen presenting cell (APC) generating a T cell response, which includes the production of anti-HER2 CD8+ cytotoxic T cells and CD4+ T cells [21]. The anti-HER2 CD4+ T cells will in turn activate a specific B cell response with consequent anti-HER2 antibody production. The second phase of the immune response occurs in the peripheral area, where cancer-specific T-cells and/or antibodies recognize HER2+ cancer cells and execute their cytotoxic effect (Fig. 1).

However, as soon as cancer-specific T cells enter the tumor microenvironment, the challenge of cancer-induced immune suppression begins. Tumors can obstacle DC maturation, trigger an inappropriate immune response such as TAA-tolerance, or facilitate the infiltration and expansion of T regulatory cells (Tregs), which correlates with poor prognosis [22, 23]. Normally, T cytotoxic activity is finely modulated by facilitating and inhibiting signals. In more detail, after binding of the T cell receptor (TCR) to the major histocompatibility complex (MHC)-antigen complex, the B7 protein on the APC binds to the T cell costimulatory molecule CD28 to promote T cell activation and survival. Inhibition of T cell activation is mediated by the subsequent upregulation of cytotoxic T lymphocyte antigen 4 (CTLA-4) on T cells, which competes with CD28 to bind B7. Further control of T cell response in peripheral tissues is regulated by the expression of PD-1 on the activated T cells. The binding of PD-1 to its programmed death-ligand 1 (PD-L1) induces an inhibitory signal that limits T cell proliferation and cell survival [24–26]. Cancer cells may overexpress surface molecules that cause T cell anergy, such as PD-L1 [27]. The spontaneous immune response to HER2+ BC may be eluded at any step of the processes involved in cancer initiation, progression, and metastatic spread. Therefore, the development of a successful cancer immunotherapy strategy in HER2+ BC has to contemplate the overcoming of all the above barriers. In accordance with the aforementioned two domains of immune response, we can divide the immunotherapeutic strategies into two groups: the first one seeks to enhance the maturation/activity of the DCs/T effector cells, and can be performed throughout the use of vaccines; the second one comprises all the modalities developed to overcome the immunosuppressive mechanisms at the tumor microenvironment level and can be mediated by immune checkpoint inhibitors and partially by enhancing the antibody-dependent cell-mediated citotoxicity (ADCC) effect of the anti-HER2 agents.

When considering anti-cancer immunotherapeutic strategies for HER2+ BC, it is important to define the inherent immunogenicity, which has some proposed surrogates such as tumor mutational burden (TMB), tumor infiltrating lymphocytes (TILs), and PD-L1 expression. Whole exome sequencing of tumor samples has uncovered a positive association in melanoma and non-small cell lung cancer (NSCLC) between response to immunotherapy and TMB [28, 29]. Higher TMB is also predictive of better clinical activity of immunotherapy in terms of objective response (OR), progression-free survival (PFS), and overall survival (OS) in colorectal cancer [30, 31] and other tumor types [29, 32, 33]. The median somatic mutation frequency of BC is 10 times lower than that of highly immunogenic tumors such as melanoma, NSCLC, bladder cancer, and colorectal cancer [9, 34]. However, there is high variability among BC subtypes, especially between hormone receptor positive (HR+) tumors and the hormone negative (HR-) ones, with these latter generally showing higher TMB [35, 36]. Also HER2 positivity in BC confers higher TMB, as a study on a HER2+ mBC patient population showed. More than one third of tumors analyzed in the study had a high TMB (> 100 mutations), which predicted a longer median overall survival (mOS) (*p* = 0.016) [37].

The second immunogenicity correlate is represented by the number of immune cells infiltrating breast cancer bed—tumor infiltrating lymphocytes (TILs), expression of a specific pre-constituted immunity. Tumors with more than 50–60% of TILs, defined as lymphocyte-predominant BC (LPBC), have relatively good prognosis even if they belong to a less favorable subtype such as TNBC or HER2+ [38]. Incidence of LPBC is 20% for TNBC, 16% for HER2 subtype, and 6% for ER-positive luminal subtype [39]. TNBC and HER2+ tumor patients with more TILs have also better outcomes with standard treatment in terms of response to neoadjuvant chemotherapy, event-free survival (EFS), and OS [38–47]. This evidence suggests potentiality of TILs as a predictive and prognostic marker in early/locally advanced HER2+ BC.

The third correlate, which is currently used as a predictive biomarker for immunotherapy in some tumors, is PD-L1 expression. The programmed death-ligand 1 (PD-L1) antigen on tumor cells (and some immune cells) interacts with PD-1 on the immune cell surface to inhibit cytotoxic T-cell activity and stimulate regulatory T-cell development, thereby extinguishing the immune response [48]. The PD-L1 expression is heterogeneous across breast cancers and is generally positively associated with the presence of TILs and aggressive molecular subtypes such as TNBC and HER2+ BC [49]. Studies including mixed populations of patients with BC in relation to the tumor subtype (mostly TNBC in the early setting), showed that higher PD-L1 expression in BC is generally associated with longer OS in early disease [50–54], higher pCR rate after neoadjuvant treatment and better EFS [53, 55, 56]. A study conducted in a pure cohort of HER2+ BC patients with locally advanced disease showed an 18% frequency of PD-L1 expression, which was positively associated with high TILs and longer OS [57]. Data suggest that PD-L1 expression may be considered a relevant prognostic and predictive factor in HER2+ BC, whose role needs to be better defined. In the following sections, evidence on anti-cancer immune response was classified by prioritizing the treatment strategy (HER2-block, immune checkpoint inhibition, anti-cancer vaccines). As it will be reflected in the heading of each respective section, evidence on HER2-block regards strategies to improve its immune-mediated mechanism, evidence on immune checkpoint inhibition is focused mostly on the advanced disease, while data on anti-cancer vaccines were more abundant for the early disease.

## Enhancing the HER2-block immune-mediated mechanism

In invasive HER2+ BC, trastuzumab represents a landmark of treatment, which has been confirmed to improve clinical outcomes across the different settings [58–60]. It is a humanized antibody that blocks HER2 signaling inducing G1 cell-cycle arrest and inhibition of PI3K/Akt pathways, which lead to apoptosis and inhibition of angiogenesis [61]. Besides this anti-trophic effect, evidence has sustained also an immune-based mechanism of trastuzumab. Throughout a human fractal crystallizable (Fc) region, trastuzumab is able to facilitate ADCC and prime target cells for attack by the immune system [62, 63]. Trastuzumab treatment is characterized by the occurrence of primary or secondary resistance in virtually all patients with metastatic disease [64]. Subsequent anti-HER2 agents have been developed in order to extend the efficacy of HER2-block beyond the resistance obstacle.

Newer agents such as lapatinib, a dual HER1/HER2 kinase inhibitor, the HER2/HER3 dimerization inhibitor pertuzumab and more recently, the panher (HER1, 2, and 4) kinase inhibitor Neratinib can postpone or overcome anti-HER2 resistance and have yielded clinical advantages when administered in combination with chemotherapy, hormone therapy, and/or another HER2 inhibiting agent [5, 6, 65]. However, pertuzumab improves only the anti-trophic effect of the HER2-block, while as it was shown by the EGF104900 study, lapatinib also amplifies the trastuzumab-induced ADCC effect [66]. Other agents or strategies have been developed in order to enhance the immune mechanism revealed during trastuzumab-mediated HER2-block (Table 1). Ado-trastuzumabemtansine (T-DM1) is an antibody-drug conjugate, composed of trastuzumab and the cytotoxic component DM1, which increases the therapeutic range of the anti-HER2 mechanism by partially overcoming the anti-HER2 resistance to other HER2-blockers. The cytotoxic component DM1 acts by a microtubule depolymerizing mechanism, which has been shown to facilitate the DC functions [7, 85–89]. Preclinical studies have investigated a possible role of T-DM1 as a potential synergic factor in combination with immune checkpoint inhibitors like anti-PD-L1 or anti-CTLA-4 [17, 18]. Proceeding from these results, the KATE II study evaluated the combination of the anti-PD-L1 atezolizumab with T-DM1, showing promising preliminary results in previously treated HER2+ mBC in terms of response rate and progression-free survival (PFS) [90].

**Table 1:** Published clinical trials of metastatic HER2 + breast cancer patients treated with immunotherapy

Name	Drug	Phase	Setting	Number of HER2-positive patients	Type of intervention	Mechanism of action	Outcomes
Diéras et al. [7]	TDM-1	III	Metastatic	991	HER2+ block enhancer	Antibody conjugate	-PFS (*p* < 0.001) -OS (*p* < 0.001)
Bang et al. [67]	Margetuximab	I	Metastatic	27	HER2+ block enhancer	ADCC stimulation against HER2 receptor	-Dose finding -Clinical benefit rate
Alsina et al. [68]	MCLA-128	I/II	Metastatic	8	HER2+ block enhancer	ADCC stimulation against HER2/HER3 receptors	-Dose finding -Clinical benefit rate
Borghaei et al. [69]	2B1	I/II	Metastatic	20	HER2+ block enhancer	NK stimulation against HER2 receptor	-Objective response rate -OS -TTP
Dirix et al. [70]	Avelumab	Ib	Metastatic	26	Immune check point inhibitor	Anti PD-L1	-Objective response rate -PFS -OS
Manguso et al. [71]	Pembrolizumab	Ib/II	Metastatic	58	Immune check point inhibitor	Anti PD-1	-Disease control (PD-L1 neg) -PFS -OS
Disis et al. [72]	NA	I/II	Metastatic	22	Vaccine	Peptide-based vaccine	Immunological response
Curigliano et al. [73]	NA	I/II	Metastatic	40	Vaccine	Recombinant HER2 protein + immunostimulant	-PFS -Objective response rate
Hamilton et al. [74]	NA	I	Metastatic	12	Vaccine	Recombinant HER2 protein + immunostimulant + Lapatinib	Immunological response
Miles et al [75]	NA	III	Metastatic	157 (15% of total patients)	Vaccine	Mucine-epithope (STn-KLH)	Overall TTP Overall OS (*p* = 0.916)
Heery et al. [76]	PANVAC	II	Metastatic	6 (12% of total patients)	Vaccine	DNA-based vaccine	Overall PFS (*p* = 0.09)
Norell et al. [77]	NA	I	Metastatic	8	Vaccine	Plasmid vector-based vaccine	Immunological response
Tiriveedhi et al. [78]	NA	I	Metastatic	3 (21% of total patients)	Vaccine	DNA-based vaccine	-Immunological response -Overall PFS (*p* = 0.011)
Morse et al. [79]	NA	I	Adjuvant/ Metastatic	7	Vaccine	Dendritic cell vaccine	-Immunological response - DFS
Emens et al. [80]	NA	I	Metastatic	1 (0.03%of total patients)	Vaccine	Anti-HER2 allogenic breast cancer cell line	Immunological response
Dols et al. [81]	NA	I	Metastatic	12 (43% of total patients)	Vaccine	Allogenic breast cancer cell lines	-Immunological response -TTP -OS
Chen et al. [82]	NA		Metastatic	22	Vaccine	Granulocyte macrophage colony-stimulating factor (GM-CSF)–secreting tumor vaccine	-Immunological response -Objective response rate -PFS -OS
Park et al. [83]	Lapuleucel-T	I	Metastatic	19	Vaccine	Dendritic cell based vaccine	-Immunological response -Objective response rate
Disis et al. [84]	NA	I	Metastatic	7	Vaccine	Autologous T-cell vaccine	-Immunological response -Objective response rate

Abbreviations: *ADCC* antibody-dependent cytotoxic cell, *NK* natural killer, *NA* not available, *OS* overall survival, *PFS* progression-free survival, *TTP* time to progression

Another strategy for exploiting the immune-mediated anti-cancer activity of anti-HER2 agents has been the optimization of their Fc in such a way that it becomes more efficacious in activating the ADCC. Margetuximab is a new generation mAb that targets the HER2 pathway and has a Fc region with an increased ability to mediate ADCC executed by effector cells such as NK cells and monocytes. A phase I trial tested this mAb in heavily pretreated HER2+ mBC patients, showing good tolerability and activity in this setting of patients [67]. The primary analysis of the SOPHIA trial, a randomized phase III trial comparing margetuximab plus chemotherapy versus trastuzumab plus chemotherapy in patients with HER2 + mBC who received a maximum of three prior lines was recently presented at the ASCO symposium. Margetuximab plus chemotherapy improved PFS (5.8 months versus 4.9, *p* = 0.033), ORR, and clinical benefit rate (CBR) compared with trastuzumab plus chemotherapy with an acceptable safety profile, similar to trastuzumab [91]. MCLA-128 is another anti-HER2 mAb developed as a bispecific IgG1 to enhance ADCC activity and overcome HER3-mediated resistance developed under classic HER2 blockers. A phase I/II study showed a good safety profile and consistent anti-tumor activity in heavily pretreated mBC patients who developed disease progression while on anti-HER2 therapies [68].

Other anti-HER2 bispecific antibodies have been developed under the optics of enhancing immune response to HER2+ cancer cells by promoting the encounter between lymphocytes and HER2+ cancer cells. A preclinical study investigated an engineered bispecific antibody for HER2 and CD16, employed to redirect CD16-expressing T lymphocytes and NK cells towards HER2+ cancer cells. The inherent results revealed the superiority of this agent compared to trastuzumab in triggering immune response [92]. A phase I/II clinical trial employed this bispecific antibody for treatment of patients with HER2+ mBC. Objective anti-tumor responses were not obtained. However, the therapy induced adaptive immune responses to both the HER2 intra-cellular domain (ICD) and extra-cellular domain (ECD) [69]. CD137 and CD3 are two T cell specific costimulatory receptors, which promote cell proliferation, survival, and activation [93]. The agonistic engagement of these receptors by bispecific mAbs which also bind to HER2 could both facilitate the encounter between HER2+ cancer cells and T lymphocytes and stimulate the activation of these latter [94]. PRS-343 is a bispecific fusion protein bridging CD137+ T cells with HER2+ tumor cells physically and functionally [95]. It is being tested in phase I trials for HER2+ solid tumors including breast cancer as a monotherapy [96]. Preclinical studies have suggested that the immunostimulatory approaches, such as agonistic anti-CD137 mAb and anti-PD-L1 mAb therapy, may be used to capitalize on the immune-mediated effects of trastuzumab [17]. There is an ongoing trial exploring the feasibility of treating HER2+ BC with a combination of PRS-343 and the anti-PD-L1 atezolizumab [97]. An additional ongoing phase I trial is evaluating a HER2-directed bispecific antibody, which binds to CD3 in order to engage T cells against HER2+ cancer cells in different HER2+ solid tumors, including BC [98]. Another possibility that has been explored is related to the use of nanobody activation immunotherapeutics against the HER2 protein. A preclinical study focused on the efficacy of a nanobody agent that selectively redirects anti-dinitrophenyl (DNP) antibodies to the surface of HER2+ BC cells, resulting in their targeted destruction by ADCC. This study showed that this agent is capable of selectively binding to HER2+ BC cells, recruiting anti-DNP antibodies to the cell surface, and activating ADCC [99].

An additional immune mechanism of trastuzumab that may be fostered for therapeutic purposes could theoretically be its ability to increase tumor infiltration by lymphoid cells [16]. The increased recruitment of TILs and their intra-tumoral expansion is expected with the combination of an IL-2 variant targeting fibroblast activation protein-alpha and trastuzumab in an ongoing phase I trial [100] (see also Tables 3 and 4).

Also other preclinical studies suggest possible combination of anti-HER2 therapy with cytokines. A study showed that a combination of interferon gamma (IFN-γ) and anti-HER2 antibody synergistically reduce tumor growth in mammary tumor models [130]. On this basis, a small study aimed at using a recombinant approach to produce an anti-HER2 single-chain variable domain fragment (scFv) and IFN-γ fusion protein, which demonstrated superior activity over the anti-HER2 antibody and was even active on tumors that were resistant to anti-HER2 antibody therapy [131].

A further strategy that has been explored to improve trastuzumab anti-cancer efficacy is labeling it with a radionuclide. A pilot study evaluated the feasibility of treating HER2+ mBC patients refractory to previous therapies with radioimmunotherapy developed by attaching the radioactive lutetium-177 (Lu-177) to trastuzumab. This study showed that the treatment was feasible and safe and could be considered for palliative treatment of HER2+ mBC in combination with standard agents [132].

Finally, besides the development of resistance, trastuzumab presents pharmacokinetic limitations because the reaching of a therapeutic concentration at the tumor site is often hampered by potential toxicities [133]. Preclinical studies have explored strategies to overcome this barrier. A cancer-selective oncolytic adenovirus was engineered to encode trastuzumab antibody chains allowing the production of monoclonal anti-HER2 antibody directly by cancer cells, which are then lysed, releasing both new virions and the Tumor-associated antigens (TAAs) for dendritic cells (DC) recognition and activation. Efficacy of this strategy in HER2-+ cancer was shown in vivo [134, 135]. Another in vitro study reported an efficient antibody delivery system for the incorporation of trastuzumab into poly (lactic-co-glycolic) acid nanoparticles (PLGA NPs) to overcome poor pharmacokinetics and low tumor penetration by the monoclonal antibody [136].

## Immune checkpoint inhibitors in advanced disease

One of the most important breakthroughs in cancer immunotherapy has been recently reached with the advent of the immune checkpoint inhibitors, which confer to cancer patients a clear survival advantage. Although initial steps have been explored with immune checkpoint inhibitors in BC, significant results are still lagging behind in HER2+ disease. However, there seems to be a strong rationale to move forward also in this direction, since the studies show that HER2+ BC is characterized by intrinsic immunogenicity. Moreover, immunotherapy is already exploited in a very effective way in HER2+ BC, since the predominant mechanism of trastuzumab is immune mediated. Clinical studies showed that higher TILs could have prognostic and predictive potential in HER2+ BC, besides the evidence of synergy with trastuzumab [38, 40, 137], which may indicate a synergic action between HER2-directed therapy and checkpoint inhibition. This has been the basis leading to the combination strategies. The anti-PD-1/PD-L1 agents act at the effector stage by re-energizing pre-existing T cells, while anti-CTL-4 agents act at the proliferation/activation stage by also probably enhancing de novo responses. A list of the most recent trials of immune checkpoint inhibitors administered in association with each other or combined with standard therapies or other immunomodulating strategies, e.g., vaccines, in HER2+ BC is shown in Table 1.

The inhibition of the PD-1/PD-L1 synapse at the periphery level has yielded promising outcomes. The programmed cell death ligand 1 can be expressed by both tumor cells and immune cells and in BC its expression correlates with hormone receptor negativity, higher histological grade, and higher TILs [138]. Most of the data on anti-PD-1/PD-L1 inhibitors in BC derive from studies carried out in TNBC patients. Several early-phase studies investigating the PD-1 inhibitor pembrolizumab and the PD-L1 inhibitors atezolizumab and avelumab as monotherapy for mTNBC have reported objective response rates (ORRs) ranging from 4 to 23% and superior outcomes in the first line and in patients having tumors that expressed PD-L1 in ≥ 1% of cells [70, 139–141]. Association with chemotherapical drugs such as nab-paclitaxel and eribuline increased the efficacy of atezolizumab in terms of ORRs in mTNBC [142, 143]. In these same set of patients, the combination of atezolizumab with nab-paclitaxel yielded also an OS advantage in the phase III trial Impassion 130, [144], while the anti-PD1 pembrolizumab in association with standard neoadjuvant chemotherapy raised the pCR rate to 60% and 80% in two different studies [145, 146]. Considering that HER2+ BC and TNBC share similarities in terms of immunogenicity correlates such as tumor mutation burden (TMB), TILs and PD-L1 expression, a significant benefit from immune checkpoint inhibitors is expected also in HER2 overexpressing BC. However, data in this setting are relatively scarce and no single agent-study has been conducted in a pure cohort of HER2+ BC patients. As we described previously, the trastuzumab mechanism of action contemplates also an immune-mediated action and synergic effect with the combination of checkpoint with HER2-directed therapy has been observed in preclinical studies [17, 18, 147].

One of the major concerns regarding anti PD/PDL1 therapies is acquired resistance. Several underlying mechanisms have been suggested. One of the most studied processes involves the JAK1/2 signaling: interferon-gamma (IFN)-JAK1/2-STAT1 pathway, which plays a key role in T-cell-mediated citotoxicity. Evidence in support of a role of IFN gamma deregulation has come from studies of next-generation sequencing (NGS) assessment in melanoma patients treated with anti PD1 agents, wherein loss of function mutations in JAK1/2 were revealed at the time of PD in patients who had been previously (NGS) evaluated and resulted wild type at the time of response. Functional studies showed that the inactivation of JAK2 is responsible of a lack of response to IFN-gamma, which translates into a resistance to T cell-mediated cytotoxicity and an advantage to the tumor cells [148]. In addition, the comparative analysis of tumor samples in another patient showed a truncating mutation of B2M, a protein involved in the transport of MHC I molecules on the cell surface. A reduced or absent MHC I expression could impair T cell recognition of tumor cells, thus favoring immune escape [148].

Findings from studies of Clustered Regularly Interspaced Short Palindromic Repeats (CRPSR) screening, which enables genome-wide interrogation of gene function, confirmed the role of PD/PD-L1 as a mechanism of immune evasion and the impairment of IFN-gamma as acquired resistance system. Interesting clues have also emerged concerning deletion of the protein PTPN2 in tumor models, which has been associated with an increased efficacy of immunotherapy by enhancing IFN gamma pathway [71]. In addition, immunocompetent mice have shown remarkable depletion of Adar1-targeting single-guide RNAs. ADAR1 is a protein belonging to the ADAR family, a group of enzymes responsible for RNA editing, which have been recently explored for their role in promoting immunotherapy resistence. The inherent findings showed that loss of ADAR1 increased tumors’ sensitivity to IFN and enhanced tumor inflammation, as shown by the massive presence of CD4+, CD8+, and NK cells [149]. A further proposed mechanism of acquired resistence is related to LNK iperexpression. LNK is a key negative regulator of JAK-STAT signaling with an important role in hematological malignancies. LNK is highly expressed in melanoma cell lines. The aberrant concentration of LNK confers a selective survival advantage to the cells by promoting cell growth and survival and impairing the IFN-JAK1/2-STAT1 pathway [150]. The aforementioned mechanisms of acquired resistance remain in need of validation in breast cancer, especially in HER2+ BC patients, for whom the pertinent literature is particularly limited. To our knowledge, only two phase I/II trials have been published thus far.

The JAVELIN study is a phase Ib trial which tested Avelumab monotherapy (a human anti-PD-L1 IgG1 mAb) in patients with pretreated mBC, unselected for PD-L1 expression and breast cancer subtype. A total of 168 patients were treated, of whom 15.5% had a HER2+ disease. At a threshold of ≥ 1% tumor cell staining positive for PD-L1, 62.5% of patients had PD-L1+tumors. Treatment was well tolerated but no OR was recorded in HER2+ patients. This may suggest that a previously treated population may not be suitable for the evaluation of anti-PD-L1 therapy in HER2+ BC, although the relatively restricted number of patients and limited statistical power should be considered in results’ interpretation [70].

In the phase Ib/II PANACEA study, pembrolizumab in combination with trastuzumab was explored in HER2+ mBC patients who had progressed while on trastuzumab. Among them, 29% had received also pertuzumab and 72% had received prior T-DM1. The 58 enrolled patients received concurrent trastuzumab with pembrolizumab. Six patients were enrolled in the phase Ib, they all had PD-L1+ HER2+ BC and no dose-limiting toxicities were recorded. The phase II portion of the study enrolled 52 patients, of whom 77% had PD-L1+ disease, the remaining were PD-L1- HER2+ BC patients. The median follow-up for PD-L1+ and PD-L1- patients was respectively 13.6 months and 12.2 months. Seven (15%) of the 46 PD-L1-positive patients achieved an objective response, and further four (8%) patients maintained a stable disease for more than 6 months as their best response, with disease control being recorded in 11 (23%) within the subgroup of PD-L1-positive patients. The mean duration of disease control was 11.1 months. By contrast, there were no objective responders in the PD-L1-negative group. The preliminary subgroup analyses of this study showing higher levels of TILs in the PD-L1 positive tumor population, suggested that immune mechanisms might be important in trastuzumab resistance and selection for patients who are PD-L1-positive. In future studies of metastatic HER2+ disease testing HER-2 blockade combined with anti-PD-1 or anti-PD-L1 drugs, the analysis of TILs is a nodal point which deserves further investigation [151].

Recently, the randomized phase II KATE2 study evaluated the addition of atezolizumab to T-DM1 in patients with locally advanced or metastatic HER2+ BC patients who received prior trastuzumab and taxane-based therapy. The primary endpoint was PFS. One hundred thirty-three patients were randomized to receive atezolizumab plus T-DM1 and 69 patients to placebo plus T-DM1. Almost half of the patients in both arms had received prior pertuzumab for metastatic BC. The primary results of the study were presented at the 2018 San Antonio Breast Cancer Symposium. Overall mPFS in months was 8.2 in the atezolizumab group vs. 6.8 in the placebo group with no statistical significance (HR = 0.82; *p* = 0.33). However, some other interesting outcomes were reported. In the atezolizumab group, patients with PD-L1+ disease had longer PFS compared to those with PD-L1-disease, while the opposite finding emerged from patients allocated to the placebo arm. In the atezolizumab group, patients whose tumors showed TILs ≥ 5% had longer PFS with respect to patients with TIL < 5% patients. This evidence was reversed in the placebo group. No differences in ORR emerged between the two treatment arms. When patients were compared with respect to PD-L1 positivity and a TIL 5% cut-off value, the same phenomena were also observed for ORR. Treatment was well tolerated in both arms. Atezolizumab plus T-DM1 did not yield a clinically significant PFS benefit. However, a suggestion of more favorable PFS and ORR were seen with the combination in patients with PD-L1+ and/or TIL ≥ 5% disease, who seemed to have a worse prognosis if not treated with atezolizumab [90].

There are several ongoing trials using PD-1/PD-L1 inhibition and/or CTLA-4 blockage in combination with standard anti-HER2 therapy for HER2+ BC. The phase II DIAmOND study is investigating the combination of PD-L1 and CTLA-4 inhibition added to trastuzumab in patients with HER2+ mBC who progressed on prior trastuzumab-based therapy. These patients receive induction with the anti-PD-L1 durvalumab, then the anti-CTLA-4 tremelimumab every 4 weeks and trastuzumab. The primary endpoint is 1-year PFS [101].

The ongoing AVIATOR TBCRC045 trial is combining a CD137 agonist with an anti-PD-L1. CD137 is expressed on activated T cells and NK cells and is upregulated by trastuzumab, which could add a synergic effect to the CD137 agonist. Forty patients with advanced HER2+ PD-L1 unselected BC will be randomized to receive vinorelbine, trastuzumab, and avelumab; vinorelbine and trastuzumab; or vinorelbine, trastuzumab, avelumab, and utomilumab, a fully human IgG2 agonist mAb that binds to the ECD of CD137. Those receiving the 2 drugs will then go on and receive the other 3 drugs, while the remaining 2 arms will discontinue therapy. Patients must have received prior trastuzumab and pertuzumab but no prior immunotherapy [102].

The NRG BR004 is an ongoing phase III double blind trial comparing paclitaxel, trastuzumab, and pertuzumab with or without atezolizumab in patients with metastatic HER2+ breast cancer. The primary outcome is PFS [103].

Two additional, interesting randomized phase III trials are ongoing to investigating the efficacy of HER2 double block plus PD-L1 inhibition in the neoadjuvant plus adjuvant setting for HER2+ BC PD-L1 unselected BC patients. The APTneo trial is comparing trastuzumab-pertuzumab double block plus chemotherapy to atezolizumab plus the double block plus a chemotherapy, which will be different for half of the patients, with respect to the non-atezolizumab cohort. Five year event-free survival is the primary endpoint [118]. The Impassion 050 is randomizing HER2+ BC patients with locally advanced disease to receive as a pre-operatory treatment the combination of atezolizumab plus trastuzumab-pertuzumab double block plus chemotherapy or the same treatment without atezolizumab. Pathological complete response is the primary endpoint [119] (see Tables 3 and 4 for an extend list of the ongoing trials).

## Vaccines in early disease

Patients affected by cancer can spontaneously harbor specific CD8+ and CD4+ T cells for TAAs [152]. Cancer vaccines might enhance these pre-existing responses and induce de novo activation. Many initial clinical trials using cancer vaccines, which included patients with advanced stages of disease, showed poor outcomes because short peptides and ineffective DC-activating adjuvants were used [153], and anti-tumor immune response might be relatively defective in metastatic cancer [154]. Subsequently, cancer vaccine development was redefined in terms of the selected TAAs and immune adjuvants and the best clinical setting in which to be exploited, by proposing the early or minimal disease stages as the most suitable scenarios [155].

Barriers to success of cancer vaccines as single peptidic agents are numerous. The TAAs bound to MHC-I on the tumor may still not be sufficiently immunogenic and expression within the tumor bed can be heterogeneous [156]. Besides using peptides [157, 158], other approaches for vaccine development include gene vectors encoding TAAs [159] and cell-based approaches such as autologous cancer cells [160], allogenic tumor cell lines [161] and DC-based vaccines [162]. A further immunizing strategy was developed by using adoptive lymphocyte therapy using lymphocytes with tumoricidal potential [163]. The possibility of successful strategies for adoptive T cell therapy beyond patients harboring pre-existing tumor-specific T cells and MHC restriction have substantially increased with the recent advances in T cell engineering through chimeric antigen receptors (CARs) enriched by costimulatory signaling domains [164].

The anti-cancer vaccine strategy may be used to enhance the immune response against HER2+ BC. The driver oncogene product, HER2, is expressed on the surface of cancer cells, where antibodies can detect it on the intact membrane. Trastuzumab’s high efficacy showed that antibodies against HER2 can yield an anti-cancer effect. However, the majority of vaccines developed for HER2+ BC treatment induce T cell responses to HER2, not antibody responses [165]. Nevertheless, vaccine formulations that elicit exclusively an anti-HER2 antibody response (with no T cell response) have been developed preclinically and also tested in the (early) clinical setting [166]. Diverse vaccine formulations have been explored for the treatment of the BC subtypes [167]. We are now mentioning and briefly discuss evidence from the main clinical trials having administered cancer vaccines in HER2+ BC patients or that included also this subgroup of BC patients (Tables 1 and 2).

**Table 2:** Published clinical trials of HER2 + breast cancer patients treated with immunotherapy, early setting

Name	Drug	Phase	Setting	Number of HER2-positive patients	Type of intervention	Mechanism of action	Outcomes
Benavides et al. [168]	NA	I	Adjuvant	151*	Vaccine	Peptide-based vaccine (E75)	-Immune response (*p* = 0.02) -DFS (*p* = 0.4 LE; *p* = 0.7 OE) -Mortality (*p* = 0.08 LE; *p* = 0.6 OE)
Carmicheal et al. [169]	NA	I	Adjuvant	18	Vaccine	Peptide-based vaccine (GP2)	-Safety -Immune response (*p* = 0.000004)
Holmes et al. [170]	NA	I	Adjuvant	15	Vaccine	Peptide-based vaccine (AE37)	Immunological response
Limentani et al. [171]	NA	I	Adjuvant	61	Vaccine	Recombinant HER2 protein + immunostimulant	Immunological response
Peoples et al. [172]	NA	NA	Adjuvant	53	Vaccine	Peptide-based vaccine (E75)	DFS (*p* < 0.19)
Mittendorf et al. [173]	Nelipepimut-S	I/II	Adjuvant	187	Vaccine	Peptide-based vaccine (E75)	DFS (*p* = 0.08)
Patil et al. [174]	NA	I/II	Adjuvant	52 (28% of all patients)	Vaccine	Peptide-based vaccine (E75)	-Immunological response -DFS (0.52)
Peoples et al. [175]; Mittendorf et al. [176]	NA	NA	Adjuvant	186	Vaccine	Peptide-based vaccine (E75)	-Immunological response -DFS (*p* = 0.04 20 months; *p* = 0.08 24 months)
Mittendorf et al. [177]	NA	II	Adjuvant	101	Vaccine	Peptide-based vaccine (GP2)	DFS (*p* = 0.43)
Clifton et al. [178]	NA	I	Adjuvant	17	Vaccine	Peptide-based vaccine (GP2)	Immunological response
Mittendorf et al. [179]	NA	II	Adjuvant	144	Vaccine	Peptide-based vaccine (AE37)	-DFS (*p* = 0.45) -Immunological response
Higgins et al. [180]	NA	I	Neoadjuvant	15 (25% of total patients)	Vaccine	Wilms’ tumor 1 (WM1) immunotherapeutic	-Objective response rate -Immunogenicity
Valdes-Zayas et al. [181]	NA	III	Adjuvant	22 (25% of total evaluated patients)	Vaccine	Anti-ganglyoside vaccine (NeuGcGM3)	Immunological response
Czerniecki et al. [182]	NA	I	Neoadjuvant	13	Vaccine	Dendritic cell based vaccine	Immunological response
Koski et al. [183]	NA	I	Neoadjuvant	27	Vaccine	Dendritic cell-based vaccine	-Immunological response
Lowenfeld et al. [184]	NA	I	Neoadjuvant	54	Vaccine	Dendritic cell-based vaccine	-Immunological response -pCR

*Patients were stratified by HER2 expression and divided into low expressors and overexpressors. Low expressors were defined as HER2/neu immunohistochemistry (IHC) 1(+) to 2(+) or fluorescence in situ hybridization < 2.0. Overexpressors were defined as IHC 3(+) or fluorescence in situ hybridization > or = 2.0 +

Abbreviations: *ADCC* antibody-dependent cytotoxic cell, *DFS* disease-free survival, *LE* low expressors, *NK* natural killer, *NA* not available, *OE* overexpressors, *OS* overall survival, *pCR* pathological complete response

### Peptide-based and other epitope-based vaccines

HER2 protein, carbohydrate antigens such as the carcino-embryonic antigen (CEA), and Mucin-1 (MUC-1) are the three most studied BC antigens for vaccine development. Spontaneous antibody response to HER2 and MUC-1 antigens in BC patients is very low. Other targets that have been explored include Wilms’ tumor 1 antigen (WT1) and gangliosides. Peptide-based vaccines have been used mostly with the granulocyte macrophage colony stimulating factor (GM-CSF) cytokine as adjuvant to enhance efficacy [185]. They are modifiable and combinable and are associated with minimal toxicity, while offering the possibility for a potential prolonged immunity, but also characterized by HLA restriction and scarce diversity [186].

#### Anti-HER2 protein vaccines

Three main peptides derived from the protein structure of HER2 have been used to develop BC vaccines. The E75 and GP2 peptides, which are HLA-A2/A3-restricted, and AE37, which is a promiscuous HLA class II binder [168–170]. These peptides are located on the extra-cellular domain (E75), transmembrane portion (GP2), and intracellular domain (AE37) of the HER2 protein. However, formulations including a mixture of peptide sequences derived from both the ECD and ICD, such as the recombinant protein dHER2 [171], or other formulations have also been developed [72].

##### E75 vaccine

Several phase I trials tested E75 vaccine with different immune-adjuvants in mBC with scarce anti-tumor activity [153]. However, interesting results were obtained in early BC. HER2 is expressed at different levels in > 75% of BC cases. A study in the adjuvant setting showed that low HER2 expressors (immunohistochemistry 1+ (IHC1+)), generally display more robust immunologic responses and possibly derive the greatest clinical benefit from the E75 vaccine [168]. Different phase I and phase II trials were conducted in the adjuvant setting, mostly for high-risk BC with any level of HER2 expression (IHC 1+ to 3+) using E75 vaccine plus GM-CSF, in patients who had completed standard treatment. A small study compared 24 vaccinated patients to 29 non-vaccinated ones. Disease-free survival (DFS) rates at 22 months were 85.7% and 59.8%, respectively (*p* = 0.19) [172]. Another phase I/II clinical trial used a similar treatment in this clinical setting by comparing 108 vaccinated patients to 79 controls. The DFS rates at 60 months were 89.7% and 80.2%, respectively (*p* = 0.08). The number of HER2+ BC patients who received trastuzumab was comparable across the two arms and, also in this subset of patients, the DFS advantage was maintained, although at a not statistically relevant extent [173]. A previous study showed that E75 vaccine plus GM-CSF were effective in raising anti-HER2 immunity in both HLA-A2+ and HLA-A3+ patients in the adjuvant setting. At a 26-month follow-up, DFS rates were 91.7% in the A2+/A3+ groups taken together and 85.2% in the control group, while at 30 months, the DFS rate was 92.3% for A3+ patients (*p* = 0.52). It is noteworthy that the HER2 positivity rate was significantly higher in the HLA-A2+ vaccinated group [174]. The combined results of two additional trials that investigated E75 vaccine plus GM-CSF in patients with high risk breast cancer added some interesting hints for future investigations [175]. A total of 101 HLA-A2+ and HLA-A3+ patients were vaccinated and compared to 85 HLA-A-/HLA-A2- non-vaccinated patients. The HER2+ BC patients constituted 25.8% of the vaccinated individuals and 28.4% of the control group. At a median follow-up of 20 months, DFS rates of the vaccinated group vs. control group were 94.4% and 85.8%, respectively (*p* = 0.04). This difference ceased to be statistically significant at a 24 months median follow-up, when DFS rates were respectively 94.3% vs. 86.7% (*p* = 0.08). Subset analyses showed that patients benefited more from the vaccine if they had HER2 IHC 1+/2+ disease (*p* = 0.04) [176]. When the follow-up was extended to 5 years, as the E75-specific immunity waned, the recurrences occurred more frequently. Therefore, an effective booster protocol was implemented on the patients who had failed to maintain a residual immunity [187]. No statistically significant differences in DFS rates were registered in HER2 IHC3+ BC patients. There are two current ongoing phase II trials trying to clarify the role of anti-HER2 vaccines in HER2-expressing BC patients, especially in relation to the concomitant use of trastuzumab. One study is testing the E75 plus GM-CSF vaccine in combination with trastuzumab in patients with high-risk, HER2 IHC 3+ BC [104] and the other one is evaluating the same combination with trastuzumab in patients with HER2 low-expressing tumors (IHC 1+ or 2+) [120]. Moreover, a phase III clinical trial using E75 plus GM-CSF is currently ongoing for the adjuvant setting of patients with low to intermediate HER2 expression BC [121].

##### GP2 vaccine

A phase I trial explored the GP2 vaccine plus GM-CSF treatment feasibility in BC after standard adjuvant treatment. Eighteen node-negative, HER2 IHC 1+ to 3+ HLA-A2+ BC patients were vaccinated (6 of them were HER2 IHC 3+). Treatment was safe and elicited anti-HER2 specific immune response [186]. A subsequent phase II study was conducted to determine the efficacy for BC patients with high-risk, HER2 IHC 1+ to 3+ disease. A total of 180 patients were randomized to receive GP2 vaccine plus GM-CSF (89 patients) or GM-CSF alone (91 patients). The 5-year DFS rate for the GP2 plus GM-CSF group vs. the GM-CSF only group, after a 34-month median follow-up, (per-treatment analysis), was not significantly different, being respectively 94% and 85% (*p* = 0.43), and if only HER2 IHC3+/FISH+ patients were considered, 100% vs. 89%, respectively (*p* = 0.08). This trial suggested that vaccination may have clinical activity, particularly in patients with HER2 IHC 3+/FISH+ BC and that this benefit is to ascribed to the vaccine, with respect to the immune adjuvant alone [177]. Another phase Ib trial tested GP2 vaccine plus GM-CSF and trastuzumab in HER2+ (IHC 3+/FISH+) BC patients. Treatment was safe and stimulated a broad immunologic response supporting further investigation of combination strategies between anti-HER2 vaccines and trastuzumab [178].

##### AE37 vaccine

AE37 vaccine plus GM-CSF was tested in the adjuvant setting of BC patients. In a phase I trial, the vaccine showed to be safe and to be able to elicit HER-2/neu-specific immune responses [170]. A randomized phase II trial was conducted to determine efficacy of this vaccine for high-risk, HER2 IHC 1+ to 3+ BC patients. Two hundred ninety-eight patients were randomized to receive AE37 plus GM-CSF vaccine vs. GM-CSF alone. Among the HER2 IHC3+/FISH+ patients, 74 in the vaccination group and 60 in the control group also received trastuzumab. The 5-year DFS rate after 25 months of median follow-up was 80.8% in the vaccinated group vs. 79.5% in the control patients (*p* = 0.7), when all patients were considered; 83.2% in vaccinated patients vs. 88.0% in controls, when HER2 IHC 3+/FISH+BC patients were considered (*p* = 0.45); 77.2% in the vaccinated ones vs. 65.7% in controls for the HER2 IHC 1+/2+ BC patients (*p* = 0.21); and 77.7% in vaccinated patients vs. 49.0% in control patients for TNBC (HER2 IHC 1+ to 2+/FISH- and HR-) (*p* = 0.12). In none of the subsets, a significant advantage in terms of DFS rate was seen. However, the study showed that the vaccine is safe and suggested that vaccination may have clinical benefit in patients with low HER2-expressing tumors, especially if TNBC [179]. The evidence available for anti-HER2 directed vaccines in the metastatic setting for HER2-expressing BC is limited. Results from the most representative studies are summarized below.

##### Anti-recombinant HER2 protein (dHER2) vaccine

A phase I study assessed a recombinant HER2 protein (dHER2) vaccine combined with the immunostimulant AS15, in the adjuvant setting, in 61 trastuzumab-naive patients with HER2+ (IHC3+/FISH+) BC. The vaccine was safe and a specific immunological response was elicited, which increased with the dHER2 dose [186]. A parallel phase I/II study assessed the vaccine in 40 metastatic HER2 IHC3+/FISH+ BC patients, who received it as first- or second-line after a documented response to maintenance trastuzumab. The treatment was well tolerated. Both humoral and cellular immunity were activated, with one patient experiencing a complete response and another one a partial response. A stable disease was reported in 10 patients [73]. A phase I trial showed that dHER2 vaccine plus AS15 could be given concomitantly with lapatinib in HER2 IHC3+/FISH+ mBC patients that were trastuzumab refractory. A total of 12 women were immunized while receiving lapatinib concurrently. The treatment was well tolerated, and anti-HER2 antibodies were induced in all patients, whereas HER2-specific T-cell response was detected in only 1 patient. There were no objective responses. One patient remained free from disease progression for 6 months. Median time to progression was 55 days, with the majority of patients progressing prior to the induction peak of anti-HER2 immune responses. However, the 300-day OS rate was 92% [74].

##### Intracellular and extra-cellular domain anti-HER2 protein vaccines

A phase I/II trial tested concurrent trastuzumab and ICD plus ECD peptide anti-HER2 vaccination in patients with HER2 IHC 3+/FISH+ mBC, who had reached a complete remission or stable disease on trastuzumab treatment. Twenty-two patients on trastuzumab therapy were vaccinated. The treatment was well tolerated and immune response was significantly boosted and maintained. At a median follow-up of 36 months, mOS was not reached. A possible synergy between trastuzumab and the vaccine was suggested [72].

##### Anti-MUC1 vaccines

MUC1 is a glycoprotein overexpressed in BC. Its epitope was conjugated with a carrier protein to form a BC vaccine, which showed promising results in early-phase trials. However, a randomized phase III trial that enrolled 1028 patients with mBC found no benefit from the vaccine in terms of time to progression (TTP) and OS. Sixteen percent of the patients in the vaccination group and 14% in the control group had HER2+ BC [75].

##### Anti-WT1 vaccines

Wilms’ tumor 1 antigen (WT1) can be overexpressed in BC [188]. A phase I trial assessed a WT1 vaccine combined with AS15 in the neoadjuvant setting for WT1+ BC. Fourteen of the 62 vaccinated patients had HER2+ BC and received concomitant trastuzumab chemotherapy. Nine of them had pCR, 4 had pPR, and 1 patient had no response. Treatment was well tolerated, but humoral response could not be elicited, probably due to the co-administration of corticosteroids [180].

##### Anti-ganglioside vaccine

N-glycolylsialic acid (NeuGc) containing gangliosides are overexpressed in many human tumors and constitute potentially immunogenic molecules [189]. A phase I trial showed that a vaccine based on the NeuGc-GM3 ganglioside was safe and immunogenic in advanced BC patients [190]. A phase III trial tested this vaccine in the adjuvant setting for BC patients. Of the 560 enrolled patients, 88 patients were randomly evaluated for immunological response, and only 22 of them had HER2+ BC. Seventeen of them randomly received the vaccine, while the other 5 received the placebo. The treatment was safe and high levels of anti-NeuGc-GM3 antibodies were detected, which was associated with a better clinical outcome [181].

##### Personalized mutanome vaccines

Personalized mutanome vaccines are a new generation of promising anti-cancer vaccines that rely on the concept according to which tumor mutations are unique for individual patients. The effective development of this therapeutical approach has become possible mainly thanks to the advent of next-generation sequencing (NGS), which is associated to dedicated bioinformatic tools in order to build a comprehensive map of tumor mutations (mutanome) and a reliable prediction of epitope-binding to MHC molecules. The process to customize a mutanome cancer vaccine needs tumor tissue and healthy tissue from the patient. Subsequently, both tumor cells and normal cells are subjected to a comparative genomic sequencing in order to find the somatic mutations and to exclude the germline mutations as possible epitopes. Once identified the somatic mutations, those with the highest likelihood to be recognized by the MHC molecules and those with a major clinical relevance are selected. After the process is completed, the epitopes generated are injected to the patient. To our knowledge, there are no published data of personalized mutanome vaccines in HER2-positive breast cancer patients. However, promising results have been reported in other cancer types. A phase I trial evaluated the safety and efficacy of personalized vaccines in untreated stage III and IV melanoma patients. Of six vaccinated patients, 4 were disease free at 25 months of follow up while 2 patients who progressed to the vaccine treatment received an anti PD1 therapy obtaining a radiological complete response. The immunological assays showed an important immune system activation against the neoepitopes which involved both CD4+ and CD8+ cells [191]. Another study conducted in 13 patients with stage III and IV melanoma tested a RNA-based poly neo-epitope showing similar outcomes: all patients developed a T cell response to the treatment and the rate of metastatic events were significantly reduced after the start of vaccination (*p* < 0.0001) which translated into a better PFS [192].

### Gene-based vaccines

The gene-based vaccine formulations use DNA structures of viruses or plasmids to deliver DNA sequences coding for TAAs. They are safe and can potentially elicit both humoral and cell-mediated immune response [193, 194].

#### Viral vector-based vaccines

##### Panvac

Viral vector-based vaccines have been used for TAAs such as HER2, p53, and MUC1, mostly in clinical trials involving metastatic BC patients [195]. PANVAC is a cancer vaccine therapy delivered through two viral vectors, which include transgenes for MUC-1, CEA, and for three human T cell costimulatory molecules [196]. This vaccine was safe in mBC patients and showed activity [197]. A phase II trial used docetaxel with or without PANVAC plus GM-CSF in mBC patients. Twenty-five patients received docetaxel and the vaccine, while 23 received standard docetaxel alone. Six HER2+ patients were included, 3 for each arm. The combination treatment of docetaxel with PANVAC resulted in a longer progression-free survival (PFS) compared to docetaxel treatment alone (7.9 vs. 3.9 months) (*p* = 0.09), but the number of HER2+ BC patients was too scarce to draw any firm conclusion [76].

#### Plasmid vector-based vaccines

Plasmid DNA can induce antibody responses to viral and non-viral antigens [198].

##### Anti-HER2 DNA vaccine

A pilot clinical trial used a plasmid vaccination with DNA encoding for HER2 protein plus low doses of GM-CSF and interleukin-2 (IL-2), in patients with HER2+ mBC, who were also treated with trastuzumab. Vaccination was well tolerated and could induce long-lasting cellular and humoral immune responses against HER2 protein [77].

##### Anti-mammaglobin-A DNA vaccine

Mammaglobin-A (MAM-A) is overexpressed in 50% of HER2+ BCs [199]. A phase I study investigated a MAM-A plasmid DNA vaccine in patients with stable mBC. A total of 14 patients were vaccinated and compared to controls. Vaccination was safe, yielded a significant increase in CD8+ T cell response, and improved PFS rate at 6 months follow-up (53% vs. 33%; *p* = 0.011). Three patients of the 14 vaccinated had HER2+ BC vs. 1 in the control group [78].

### Whole-cell vaccines

These vaccine formulations can use tumor cells, DCs, or T lymphocytes [79].

#### Tumor cell-based vaccines

Vaccine strategies based on direct application of whole tumor cells or cell extracts are polyvalent immunization strategies [200], which have shown a relatively poor immunogenic potential [201]. They are prepared by irradiating allogeneic or autologous cancer cells or cell lysates [202, 203] and their immunogenicity could be increased by engineering tumor cell lines to secrete GM-CSF [204, 205] and/or by combination with chemotherapy [206, 207].

A phase I study investigated a treatment with cyclophosphamide (CY), doxorubicin (DOX), and an anti-HER2 GM-CSF secreting allogenic BC cell line vaccine. This study enrolled 28 patients with stable mBC, of whom only 1 had HER2+ BC. The treatment was safe and bioactive when given alone or after low-dose CY and DOX [80]. Another phase I study treated mBC patients with a vaccine of an allogeneic HER2+ BC cell line genetically modified to express B7-1 costimulatory protein, in association with GM-CSF. Thirty pretreated women were vaccinated, of whom 12 were with a HER2+ disease. Prolonged stable disease was observed in 4 patients but no objective response was registered. Treatment was well tolerated [81].

Another study investigated the feasibility of a treatment with cyclophosphamide, trastuzumab, and a vaccine of an allogeneic HER2+ BC GM-CSF secreting cell line in 20 patients with HER2+ mBC. The treatment was safe and yielded considerable clinical benefits in terms of OR, PFS, and OS [82].

#### Dendritic cell-based vaccines

It is one of the most relevant approaches in the field of BC vaccine development. Autologous DCs are loaded in vitro with cancer antigens using different techniques such as facilitating antigen presentation with synthetically developed peptides, antigens derived from killed autologous/allogeneic BC cells [208] or antigens isolated from cancer stem cells [209]. TAAs presence on DC surface can also be obtained by physically fusing them with cancer cell lines [210, 211]. Her2 protein antigens have been widely used in DC vaccines. A phase I study investigated a DC vaccine loaded with a HER2 antigen called *Lapuleucel-T* to treat patients with HER2+ mBC. APCs were activated in vitro with a recombinant fusion protein consisting of ICD and ECD sequences of HER2 linked to GM-CSF. Eighteen patients were treated with a resulting good tolerability. Significant cell-mediated anti-HER2 immune response was induced. Clinical benefit in terms of tumor response was recorded [83]. Another phase I study investigated an anti-HER2 DC-based vaccination on 13 patients with HER2 IHC3+ DCIS BC in the neoadjuvant setting showing specific activation of T cells, accumulation of T and B lymphocytes in the breast, and induction of tumor-lytic antibodies [182]. Subsequently, another group of study conducted two phase I clinical trials in the neoadjuvant setting for patients with HER2+ DCIS BC using a DC-based vaccine pulsed with a mixture of ICD and ECD HER2 peptides. In the first study, 27 HER2 IHC3+ BC patients were vaccinated. The first analysis showed the induction of a potent type I immunity, suggesting potential activity at a clinical level [183]. A subsequent analysis showed that the vaccine could eliminate HER2 expression in 50% of patients [212]. The second trial treated 54 patients, of whom one third had HER2 IHC2+ and two thirds had HER2 IHC3+. The vaccination was safe and immunogenic. For all the patients who achieved pCR, an anti-HER2 T-cell response was recorded in the sentinel lymph node [184]. A previous small study tested an anti-HER2 DC-based vaccine in the adjuvant setting for 7 women with high risk invasive HER2+ (IHC2+ to 3+/FISH+) BC, which had undergone standard treatment (± trastuzumab). At a 5-year follow-up, 6 patients had measurable anti-HER2 antibodies and all patients were alive [79]. A phase II trial testing efficacy in HER2+ mBC patients of vinorelbine plus an anti-HER2 DC vaccine plus tratuzumab in association with GM-CSF is ongoing [105] (see also Tables 3 and 4).

**Table 3:** Ongoing trials with immunotherapy in HER2 + metastatic breast cancer patients

	Drug	Phase	Setting	Status	Type of intervention	Mechanism of action
NCT02492711 [91]	Margetuximab	III	Metastatic	Completed	HER2-block enhancer	Increased ADCC effect
NCT03330561 [96]	PRS-343	I	Metastatic	Recruiting	Immune costimulatory	T-Cell activation
NCT03650348 [97]	PRS-343+ Atezolizumab	I	Metastatic	Recruiting	Immune costimulatory+ Immune check point inhibitor	T-Cell activation+ Anti PD-L1
NCT02829372 [98]	NA	I	Metastatic	Recruiting	Immune costimulatory (anti HER2/ CD-3 antibody)	ADCC + T-Cell activation
NCT02627274 [100]	RO6874281^⁞^	I	Metastatic	Recruiting	Immune costimulatory	ADCC + T-Cell activation
DIAmOND [101]	Durvalumab+ Tremelimumab+ Trastuzumab	II	Metastatic	Recruiting	Immune check point inhibitor	Anti PD-L1 + Anti-CTLA-4
NCT03414658 [102]	Avelumab + Utomilumab + Vinroelbine+ Trastuzumab	II	Metastatic	Recruiting	Immune costimulatory+ Immune check point inhibitor	T-Cell activation+ Anti PD-1
NCT03199885 [103]	Paclitaxel + Trastuzumab + Pertuzumab + Atezolizumab	III	Metastatic	Recruiting	Immune check point inhibitor	Anti PD-L1
NCT02297698 [104]	Nelipepimut+ Trastuzumab	II	Metastatic	Active, not recruiting	Vaccine	Peptide-based vaccine (E75)
NCT00266110 [105]	therapeutic autologous dendritic cells+ Trastuzumb + Chemotherapy	II	Metastatic	Completed	Vaccine	DC vaccine
NCT02713984 [106]	Anti-HER2 CAR-T	I	Metastatic	Recruiting	Vaccine	CAR-T cells
NCT03125928 [107]	Pertuzumab + Trastuzumab + Atezolizumab	IIA	Metastatic	Recruiting	Immune check point inhibitor	Anti PD-L1
NCT02605915 [108]	Atezolizumab+ Pertuzumab + Trastuzumab + or Atezolizumab+TDM-1	IB	Early and metastatic	Active, not recruiting	Immune check point inhibitor	Anti PD-L1
NCT03417544 [109]	Atezolizumab+Pertuzumab + Trastuzumab +	II^†^	Metastatic	Recruiting	Immune check point inhibitor	Anti PD-L1
NCT00343109 [110]	NA	II	Metastatic	Active, not recruiting	Vaccine	ICD peptide-based vaccine
NCT02547961 [111]	NA	I/II	Metastatic	Completed	Vaccine	CAR-T cells
NCT03364348 [112]	Utomilumab + TDM-1 or trastuzumab	I	Metastatic	Recruiting	Immune costimulatory	T-Cell activation
NCT01922921 [113]		I/II	Metastatic	Active, not recruiting	Vaccine	ICD peptide-based vaccine
NCT03032107 [114]	Pembrolizumab+ TDM-1	I	Metastatic	Recruiting	Immune checkpoint inhibitor	Anti PD-1
NCT00436254 [115]	NA	I	Early and metastatic	Active, not recruiting	Vaccine	pNGVL3-hICD vaccine
NCT00194714 [116]	NA	I/II	Metastatic	Active, not recruiting	Vaccine	Her2+ peptide vaccine
NCT03740256 [117]	NA	I	Metastatic	Not yet recruiting	Vaccine	Adenovirus-specific cytotoxic T lymphocytes (HER2-AdVST), in combination with intra-tumor injection of CAdVEC, an oncolytic adenovirus

^⁞^mAbdirected against fibroblast activation protein-alpha (FAP) linked to an engineered, variant form of interleukin-2 (IL-2v)

^†^Only HER2+ patients with brain metastasis

Abbreviations: *ADCC* antibody-dependent cytotoxic cell, *CAR* chimeric antigen receptor, *DC* dendritic cell, *ICD* intracellular domain, *mAb* monoclonal antibody, *NA* not available

**Table 4:** Ongoing trials with immunotherapy in HER2 + breast cancer patients, early setting

	Drug	Phase	Setting	Status	Type of intervention	Mechanism of action
NCT03595592 [118]	Atezolizumab+ Pertuzumab+ Trastuzumab+ Chemotherapy	III	Early	Recruiting	Immune check point inhibitor	Anti PD-L1
NCT03726879 [119]	Atezolizumab+ Pertuzumab+ Trastuzumab+ Chemotherapy	III	Early	Recruiting	Immune check point inhibitor	Anti PD-L1
NCT01570036 [120]	Nelipepimut+ Trastuzumab	II	Early‡	Completed	Vaccine	Peptide-based vaccine (E75)
NCT01479244 [121]	Nelipepimut+ Sagramostim	II	Early‡	Completed	Vaccine	Peptide-based vaccine (E75)
NCT02605915 [108]	Atezolizumab+ Pertuzumab + Trastuzumab + or Atezolizumab+TDM-1	IB	Early and metastatic	Active, not recruiting	Immune check point inhibitor	Anti PD-L1
NCT03321981 [122]	MCLA-128	II	Early	Recruiting	HER2-block enhancer	ADCC stimulation against HER2/HER3 receptors
NCT03747120 [123]	Pembrolizumab+ Paclitaxel+ Pertuzumab + Trastuzumab	II	Early	Recruiting	Immune check point inhibitor	Anti PD-1
NCT01355393 [124]	NA	I/II	Early	Active, notrecruiting	Vaccine	Peptide vaccine
NCT00436254 [115]	NA	I	Early and Metastatic	Active, not recruiting	Vaccine	pNGVL3-hICD vaccine
NCT03742986 [125]	Nivolumab + docetaxel+ Pertuzumab+ Trastuzumab	II	Early	Not yet recruiting	Immune checkpoint inhibitor	Anti PD-1
NCT03387553 [126]	NA	I	Early	Recruiting	Vaccine	DC Vaccine
NCT03630809 [127]	NA	II	Early	Recruiting	Vaccine	DC vaccine
NCT03620201 [128]	NA	I	Early	Recruiting	Immune checkpoint inhibitor	Anti PD-L1/TGF-Beta TRAP.
NCT03820141 [129]	Durvalumab + Trastuzumab+ Pertuzumab	II*	Early	Not yet recruiting	Immune checkpoint inhibitor	Anti PD-L1

^‡^Only patients with HER2 1+ and 2+ expressing tumors

*Only HER2-enriched patients

Abbreviations: *ADCC* antibody-dependent cytotoxic cell, *CAR* chimeric antigen receptor, *DC* dendritic cell, *ICD* intracellular domain, *mAb* monoclonal antibody, *NA* not available

#### Autologous T cell-based vaccine

There are very few data on the treatment of HER2+ BC with vaccines based on autologous T cells.

A small pilot study on only one patient with HER2+ mBC tested an adoptive transfer of autologous HER2-specific T-lymphocytes, which resulted unable to penetrate into the solid metastases. However, disseminated tumor cells in the bone marrow disappeared after the completion of the treatment [213]. Another phase I trial investigated the feasibility of a vaccine with HER2 primed autologous T cells in 7 patients with HER2+ treatment refractory mBC. The treatment was feasible and clinical responses were observed in 43% of patients. Lower number of T-regulatory (T-reg) cells in peripheral blood prior to infusion (*p* < 0.001), higher level of HER2 specific T-cells in vivo (*p* = 0.030), and development of diverse clonal T-cell populations (*p* < 0.001) were positively associated with response [84]. A further step in the autologous T cell vaccine strategy has started to be explored recently by using T lymphocytes genetically engineered to express chimeric antigen receptors (CAR-Ts). CAR-T cell therapy is currently being investigated in a phase I study enrolling patients with relapsed or refractory HER2+ solid tumors (breast cancer, ovarian cancer, lung cancer, gastric cancer, colorectal cancer, glioma, pancreatic cancer) [106].

Currently ongoing trials of immunotherapy in HER2+ BC are listed in Tables 3 and 4.

## Discussion

The overall cumulated evidence analyzed in this review showed that the modulation of the immune system for the treatment of HER2+ BC is a promising path. Trastuzumab’s passive ability to exploit immune response for its action can be enhanced and partially redirected in an advantageous way by strategies that refine its structure or by combining it with other agents in such a way that synergy is yielded in activating immune response. Approaches that addressed experimentally the modification of trastuzumab structure include the conceptualization of T-DM1, which facilitates DC’s functions; margetuximab, a mAb that increases the ACCD; and the bispecific mAb MCLA-128, capable of enhancing both ACCD and the anti-trophic effect. Studies showed that the ACCD effect of trastuzumab can also be amplified by combining it with lapatinib. Other bispecific mAbs were engineered with the intent of promoting the encounter between HER2+ BC cells and lymphocytes, while facilitating the activation of these latter. Such mAbs include formulations able to bind on one side the HER2, and on the other side the T cell’s and/or NK cell’s specific receptors or costimulatory molecules such as CD16, CD 137, and CD3. These strategies showed efficacy in preclinical and early clinical trials, some of which were ongoing at the time this review was thought and conceived. Additional approaches are being investigated mostly in preclinical studies. In vitro studies showed that nanobody agents can selectively redirect pre-existent antibodies towards HER2+ BC cells inducing ACCD. Other studies suggest a possible advantage in terms of an increase in TILs and immune response when combining anti-HER2 therapy functionally and/or structurally with cytokines such as IL-2 or IFN-γ. All these modalities offer a wide landscape where the immune-mediated mechanism of anti-HER2 agents can be further extended. Moreover, on a parallel path, the generic activity of trastuzumab can also be enhanced using other modalities, such as association with chemotherapy, labeling it with radionuclides or by oncolytic viruses or specific delivery systems that facilitate accumulation at the tumor site. However, as the aforementioned studies suggest, the most promising approach for capitalizing on the immune-mediated mechanism of anti-HER2 agents is the combination with other agents that induce/release an anti-cancer immune response utilizing a different mechanism, aiming at a synergic effect. Combination strategies may possibly act on the imbalance caused by the lower expression of PD-L1 or lower representation of TILs, enhancing cancer immunogenicity and increasing clinical benefit. Preclinical studies showed an increased activity by the joint action of HER2-block and one of the latest breakthroughs in cancer immunotherapy represented by immune checkpoint inhibition, warranting a further assessment in HER2+ BC patients. Hence, ongoing studies on HER2+ mBC patients are evaluating the efficacy of associations of enhanced HER2 blockers such as T-DM1 or bispecific mAbs with anti-PD-L1 mAbs such as atezolizumab. Immune checkpoint inhibiting mAbs have shown very promising results especially as upfront treatment in mTNBC patients with PD-L1+ tumors. Accordingly, based on the encouraging immunogenicity correlates in HER2+ BC, such as high TILs and high PD-L1 expression, also this group of patients was included in clinical trials designed to determine the efficacy of immune checkpoint inhibition of mixed BC subtype populations. In one of these clinical trials, the anti-PD-L1 avelumab did not yield any objective response in PD-L1+ HER2+ mBC patients pretreated with trastuzumab and T-DM1. However, it is important to highlight the fact that these HER2+ BC patients did not receive a concomitant HER2-block during the avelumab treatment, which could have been brought to a different result. In fact, another clinical study also included heavily pretreated HER2+ mBC patients who had progressed to trastuzumab and T-DM1, but HER2-block was maintained with trastuzumab while concurrently giving the anti-PD-L1 pembrolizumab, showing higher ORR (15% of patients had a partial response) and PFS in patients with anti-PD-L1+ tumors who received the combination compared to those with the same characteristics who received trastuzumab alone. Since the patients enrolled were trastuzumab resistant, the responses were mainly due to pembrolizumab. These two trials taken together suggest that anti-HER2+ should be continued when using the immune checkpoint inhibitors and that the heavily pretreated patients may not represent the most suitable setting to use the combination. The preliminary results of a more recent study including only HER2+ mBC patients who had progressed on trastuzumab +/− pertuzumab and who were randomized to receive T-DM1 plus atezolizumab or T-DM1 alone, showed that the combination yields an advantage in terms of ORR and PFS when tumors are PD-L1+. Even though very few data are currently available concerning HER2-positive breast cancer, response rates in the metastatic setting reach 15% and are thus fairly comparable to the 4 to 23% range recorded for TNBC. These results warrant the design of clinical trials that allocate HER2+ mBC patients to treatment on the basis of specific biomarkers and the use of an even broader range of combination strategies. Ongoing trials are investigating even more extensive agent associations in HER2+ mBC patients, combining anti-PD-L1 (durvalumab) plus anti-CTLA-4 (tremelimumab) plus trastuzumab or a compound treatment with vinorelbine plus trastuzumab plus avelumab plus utomilumab, a CD137 agonist mAb. Data on anti-HER2 agents, immune checkpoint inhibitors, or their combination refers mostly to the pretreated HER2+ mBC. However, recently two ongoing trials, the APTneo and Impassion 050, started the enrollment of HER2+ BC PD-L1 unselected patients for the neoadjuvant setting and are comparing atezolizumab plus double block with trastuzumab and pertuzumab plus chemotherapy to double block plus chemotherapy alone. Interesting results are expected in terms of pCR and event-free survival. On the basis of the underlying biological mechanism, immune checkpoint inhibition seems to find a stronger rationale for clinical use when macroscopic disease is present, which would mean in a locally advanced curable disease or a metastatic condition. Moreover, clinical trials suggest higher efficacy if the treatment is given upfront, while a concomitant HER2 block is applied. Probabilities based on evidence point to a future standard treatment consisting of the combination of immune checkpoint inhibitors and anti-HER2 agents with enhanced immune-mediated mechanism to be used as early as possible when HER2+ BC is detected. In addition, considering the high immunogenicity of HER2+ tumors and the preferable settings in which immunotherapy could be employed (presence of tumor, early lines in metastatic disease), HER2+ breast cancer may represent a fertile soil (i.e., availability of tumor tissue, fast clinical impact) also to test newer immunotherapies agents and to expand the knowledge of possible mechanisms to overcome immunotherapy resistance. As mentioned before, several mechanisms are involved in determining resilience to immunotherapy, but emerging data show that new frontiers of immunotherapy could soon be opened. We discussed the role of IFN pathway impairment as a main mechanism of acquired resistance to check point inhibitors. Therefore, restoring the immune sensitivity operated by IFN could be a viable process to overcome resistance. A possible target could be represented by agonist of stimulator of interferon genes (STING) which is a cytosolic receptor sensible to tumor-derived DNA. The presence of tumor-derived DNA enhances ligand production of STING and its activation. Once activated, STING signaling pathway produces IFN I and proinflammatory citokines triggering a potent anti-tumor response correlated with increased activation of DC and TCD8+ cells in vivo [214]. Given the STING agonist’s potential to increase anti-tumor immunogenicity and to induce an adaptive immune response, several agents are now being tested in clinical trials. Another possible resource against immune resistance is to enhance endogenous T cell functions or to transplant antigen specific T cells via ex vivo expansion of TILs [215]. Especially the latter could represent a promising target in HER2+ breast cancer if we consider the relatively high levels of TILs that can be found in those patients. Gathering evidences are focusing on tumor microenviroment as a key player in determining acquired resistance to immune check point inhibitors. More specifically, the presence of myeloid-derived suppressor cells (MDSCs) seem to reduce sensibility to immunotherapy [216]. Indeed, inactivation or modulation of MDSCs could help to restore immune sensitivity. PI3Kγ is a macrophage kinase which promotes a transcriptional signaling enhancing immunosuppression and tumor growth [217]. It is highly expressed in myeloid cells and in preclinical models mice affected by different types of tumor lacking PI3Kγ or treated with selective PI3Kγ inhibitors showed tumor regression and a restored sensibility to checkpoint inhibitors [218].

Regarding the possible advantages in radically operated patients with no macroscopic disease, the immune checkpoint inhibition by blocking the PD-1/PD-L1 synapse does not seem to have a rationale as an adjuvant treatment, at least not as a monotherapy. However, a large amount of evidence from diverse clinical studies has shown that HER2 block with trastuzumab can confer a survival advantage in the adjuvant setting for HER2+ BC patients. This advantage seems due to the anti-trophic effect of trastuzumab since it can be significantly increased if block is doubled with the addition of pertuzumab [219]. To the best of our knowledge, no evidence has come from and no studies are currently ongoing which investigate a possible advantage in the adjuvant setting by enhancing mechanisms of trastuzumab action other than that immune-mediated.

When a patient diagnosed with early HER2+ BC undergoes radical surgery, she/he can be considered in a macroscopically disease-free status. At the moment of tumor removal, the immune response process that had previously started and developed while the cancer mass was growing is suddenly “frozen”. However, this acquired immune response represents in its entirety a failure with respect to the task of stopping tumor growth, considering the fact that the tumor had become macroscopic. Nevertheless, in an early phase, the immune response against HER2+ BC, compared to a metastatic disease, has been only partially escaped and most importantly, the cancer-related immunosuppressive mechanisms become increasingly predominant as tumor burden rises and distant disease diffusion become more evident Therefore, in a patient with no macroscopic disease, the immune modulating strategy should point at redirecting the early immune response towards theoretical diffused cells that escaped it in a first moment. This target seems more reachable by strategies that enhance the early phases of immune activation, by using anti-HER2-specific vaccine therapies which, with different modalities, can amplify de novo activation and fortify the pre-existent immune response. The vaccine therapy for all HER2+ (IHC3+/FISH+) BC settings has to contemplate the association with HER2-block, given the established efficacy of the treatment. Interestingly, anti-HER2 vaccines tend to be more active in HER2 IHC1+/2+ BC (low and intermediate expressors), where anti-HER2 agents are not efficacious given with the standard modalities. And here, according to our opinion, an important question that should be addressed in future studies is whether anti-HER2-block, e.g., trastuzumab and/or pertuzumab, could work also for patients with low to intermediate HER2-expressing tumors if combined to anti-HER2 vaccination, especially in the adjuvant setting. Regarding this HER2 IHC1+ to 2+/FISH- BC patients, in studies conducted thus far, combination partners were sought in the field of immunotherapy agents or in classic therapies such as chemotherapy. Most of the data on the clinical benefits from anti-HER2 BC vaccines derive from studies conducted in the adjuvant setting, using HER2 peptide vaccines in association with GM-CSF as immune-stimulant. Preclinical studies with E75, GP2, and A37 vaccines showed their ability to activate a cell-mediated anti-HER2 immune response in most of the cases. Their use in a clinical setting often yielded a numerically higher DFS; however, in most of the cases, this advantage was not statistically and clinically significant. These studies showed that anti-HER2 peptide vaccines generate a stronger immune response in HER2 IHC1+/2+ BC, and when used for HER2 IHC3+/FISH+ BC, HER2-block should be maintained. Other anti-HER2 peptide vaccines containing more epitopes, from both ICD and ECD of HER protein, were tested mostly in metastatic disease with a low degree of action recorded. In order to increase the efficacy of this group of vaccines, more appropriate epitopes can be selected and different doses or types of immune adjuvant can be tested in the future. Besides peptides, other monovalent vaccines were developed for HER2+ BC using other cancer-specific antigens such as MUC-1 vaccine in metastatic setting, WT1 vaccine for neodjuvant patients and gangliosides in adjuvant treatment, with some encouraging results. Also, gene vaccines were used in trials that included mostly HER2+ mBC patients. The selected genes were mostly represented by HER2, MUC-1, CEA, and MAMM-A. In vitro results were promising, the vaccines were safe in the clinical setting and some benefit in terms of PFS was recorded. More elaborate and expensive vaccine strategies have been developed using whole tumor or immune system cells, which can be engineered to adapt to specific biological mechanisms of action. However, anti-HER2 vaccines developed by engineering HER2+ BC cell lines failed to yield response in the metastatic setting. The most widely used DC-based vaccines were loaded with HER2 epitopes using different modalities and tested for the treatment of HER2+ BC in the metastatic, adjuvant, and neoadjuvant settings with mixed results. In all the cases, vaccine was combined as indicated with trastuzumab and/or chemotherapy. Moreover, anti-HER2 DCs showed activity in the pre-operatory setting of HER2+ in situ ductal carcinoma (DCIS) patients, opening a possible new scenario in this fraction of patients. Lastly, also anti-HER2 directed autologous T cells were used as a vaccine for m HER2+ BC in a few patients. Treatment seems feasible, but efficacy should be further investigated. The general emerging pattern is that anti-HER2 vaccine cancer therapy has a weaker rationale in a late-disease setting, when cancer is metastatic and tumor burden is high. As we could observe from the results of the different studies, the failure of vaccine therapy was more frequent in the metastatic setting or whenever tumor burden was very high. This was probably due to the fact that in an advanced phase, cancer cell clones are more diverse and the escape mechanisms from the immune system have already been established and include a pronounced immunosuppressive nature of the tumor microenvironment. The development of vaccines for the later phases of disease should consider the contemporary use of more antigens, while combining vaccination with additional anti-cancer treatment strategies such as immune checkpoint inhibitors, which have a strong rationale for clinical use not only in metastatic disease but whenever a macroscopic tumor is present, including the early and locally advanced phases. Moreover, most of the developed vaccines induce an anti-HER2 T cell response, but results from a phase I trial of Berzofsky showed that DC vaccines against HER2+ BC can be designed to elicit a humoral response resulting in the production of specific anti-HER2 antibodies, which act by a different mechanism with respect to trastuzumab, being able to overcome resistance towards this agent. This vaccine was tested in a phase I trial to treat patients with advanced HER2+mBC who had failed all other therapies, and showed preliminary evidence of clinical benefit, including complete response, partial response, and stable disease lasting at least 6 months, and decrease in circulating tumor cells [166]. Therefore, both categories of cancer vaccines (targeting both arms of the adaptive immune system) can be translated into higher efficacy and better clinical outcomes.

In a wider view, definitive predictive indicators are still to be established and many possibilities are being sought in TMB, CD8+ T cell density, and oncogenic mutations [220]. These factors could also be used to better stratify patients for future studies. In HER2+ BC, TILs are factually a pre-existing anti-tumor immunity and have predictive and prognostic potential. Identifying the patient subset that benefits the most from immunotherapy with checkpoint inhibitors and/or vaccines remains a significant challenge. Studies have shown contrasting results regarding PDL1 expression as a predictive factor in breast cancer.

## Conclusions

In recent years, the body of evidence on immunotherapy in HER2+BC has grown immensely. When globally considered, the available data encourage further investigation. This is partly due to the overall encouraging results obtained thus far. At the same time, further research is inspired by an increasing awareness concerning the *plethora* of still unclarified etiopathogenetic mechanisms and unexploited potentials. In the next future, the immune-mediated mechanism of anti-HER2 agents may be further amplified in terms of the ability to induce ADCC or to put lymphocytes into action. In addition, immune checkpoint inhibition may offer space for additional optimization, especially by establishing reliable biomarkers of efficacy. Vaccine therapy may be developed in such a way that it could act also in a later phase of the history of the tumor immune system interaction, in order to reduce tolerance acquisition by cancer cells. Moreover, as we could observe, a specific advantage of anti-HER2 vaccines for BC treatment is that they can yield clinical efficacy especially when HER2 protein presents an intermediate level of expression, which could extend the ground of action for an HER2-directed immunotherapeutic strategy. The anti-HER2 vaccine spectrum of action could also be expanded by using strategies to develop vaccines that elicit at the same time cell-mediated and humoral responses. Nevertheless, the most relevant point is the prospective of finding combination strategies that translate into a synergic enhancement of anti-cancer immune response. This aim may be pursued especially by combining together two or more immune modulating strategies that act with distinct biological mechanisms, such as HER2-block, immune checkpoint inhibition, and anti-cancer vaccination. These associations could increase the magnitude of efficacy in a given HER2+ BC setting and/or extend the landscape of treatment applicability in terms of biological characteristics, such as anti-HER2 vaccine-based immunotherapy plus trastuzumab for HER2 IHC1+/2+ BC or anti-HER2 vaccines for HER2 IHC3+ DCIS; or expand treatment possibilities in terms of clinical setting, such as the use of anti-HER2 vaccines in the metastatic/neoadjuvant setting or immune checkpoint inhibition in the early disease. Moreover, the combination strategies should contemplate also classical treatments such as chemotherapy, radiation therapy, or other local approaches, which should maintain their established role in HER2+ BC and possibly give additional benefit when integrated with immune-mediated therapies. All the aforementioned strategies should be conceptualized dynamically and functionally inside the larger blueprint of rapidly developing cancer treatment *paraphernalia*, embodying further immune modulating tactics or therapies which act on a gradually expanding core of cancer hallmarks.

## Data Availability

Not applicable as no datasets were generated or analyzed.

## Abbreviations

APC: Antigen presenting cell
BC: Breast cancer
CARs: Chimeric antigen receptors
CTLA4: Cytotoxic T lymphocyte-associated antigen 4
DCs: Dendritic cells
ECD: Extra-cellular domain
EFS: Event-free survival
GM-CSF: Granulocyte macrophage colony-stimulating factor
HER2+: Human epidermal growth factor 2 positive
HLA: Human leukocyte antigen
HR: Hormone receptors
ICD: Intra-cellular domain
IFN-γ: Interferon gamma
IgG: Immunoglobulin G
IHC: Immunohistochemistry
LPBC: Lymphocyte-predominant breast cancer
mAb: Monoclonal antibody
NGS: Next-generation sequencing
NK: Natural killer
OR: Objective response
ORRs: Objective response rates
OS: Overall survival
PD-1: Programmed cell death protein 1
PD-L1: Programmed cell death-ligand1
PFS: Progression-free survival
scFv: Single-chain variable domain fragment
TAAs: Tumor-associated antigens
TCR: T cell receptor
T-DM1: Ado-trastuzumab emtansine
TILs: Tumor infiltrating lymphocytes
TKI: Tyrosine kinase inhibitor
TMB: Tumor mutation burden
TNBC: Triple negative breast cancer
Tregs: T regulatory cells
